# Bp-13 PLA_2_: Purification and Neuromuscular Activity of a New Asp49 Toxin Isolated from *Bothrops pauloensis* Snake Venom

**DOI:** 10.1155/2015/826059

**Published:** 2015-02-19

**Authors:** Georgina Sucasaca-Monzón, Priscila Randazzo-Moura, Thalita Rocha, Frank Denis Torres-Huaco, Augusto Vilca-Quispe, Luis Alberto Ponce-Soto, Sérgio Marangoni, Maria Alice da Cruz-Höfling, Léa Rodrigues-Simioni

**Affiliations:** ^1^Department of Pharmacology, Faculty of Medical Sciences, State University of Campinas (UNICAMP), 13083-881 Campinas, SP, Brazil; ^2^Department of Biochemistry and Tissue Biology, Institute of Biology, State University of Campinas (UNICAMP), 13083-365 Campinas, SP, Brazil; ^3^Multidisciplinary Research Laboratory, São Francisco University, 12916-350 Bragança Paulista, SP, Brazil

## Abstract

A new PLA_2_ (Bp-13) was purified from* Bothrops pauloensis* snake venom after a single chromatographic step of RP-HPLC on *μ*-Bondapak C-18. Amino acid analysis showed a high content of hydrophobic and basic amino acids and 14 half-cysteine residues. The N-terminal sequence showed a high degree of homology with basic Asp49 PLA_2_ myotoxins from other* Bothrops *venoms. Bp-13 showed allosteric enzymatic behavior and maximal activity at pH 8.1, 36°–45°C. Full Bp-13 PLA_2_ activity required Ca^2+^; its PLA_2_ activity was inhibited by Mg^2+^, Mn^2+^, Sr^2+^, and Cd^2+^ in the presence and absence of 1 mM Ca^2+^. In the mouse phrenic nerve-diaphragm (PND) preparation, the time for 50% paralysis was concentration-dependent (*P* < 0.05). Both the replacement of Ca^2+^ by Sr^2+^ and temperature lowering (24°C) inhibited the Bp-13 PLA_2_-induced twitch-tension blockade. Bp-13 PLA_2_ inhibited the contractile response to direct electrical stimulation in curarized mouse PND preparation corroborating its contracture effect. In biventer cervicis preparations, Bp-13 induced irreversible twitch-tension blockade and the KCl evoked contracture was partially, but significantly, inhibited (*P* > 0.05). The main effect of this new Asp49 PLA_2_ of* Bothrops pauloensis *venom is on muscle fiber sarcolemma, with avian preparation being less responsive than rodent preparation. The study enhances biochemical and pharmacological characterization of* B. pauloensis *venom.

## 1. Introduction

Phospholipase A_2_ belongs to an expanding superfamily of enzymes that catalyzes ester bond hydrolysis at the sn-2 position of 1,2-diacyl-sn-3-phosphoglycerides and generates arachidonic acid. Depending on the molecular taxonomy, intracellular and secretory PLA_2_s are currently classified in six to twelve groups [1]. Secretory PLA_2_s are enzymes of 13–18 kDa with 5–8 disulfide bonds whose activity requires millimolar Ca^2+^ concentration [2].

Despite the variety of the local and systemic pathophysiological effect, such as myotoxicity, neurotoxicity, anticoagulation, hemolysis, hypotension, platelet aggregation inhibition, and bactericidal and proinflammatory activities, PLA_2_ groups show highly conserved molecular regions and similar three-dimensional structure [3]. The majority of such local and systemic effects caused by* Bothrops* sp. envenomation is often due to the PLA_2_ activity [3–5]. Although generally the neurotoxic effects are unnoticed clinically, they can be observed during* in vitro* experiments and are frequently associated with PLA_2_ of bothropic venoms [6–14].

*B. pauloensis *is found in humid and cool regions in the central and Southwest of the state of São Paulo [15, 16] and in seasonally dry savannas of the Brazilian Cerrado [17]. From the 292 notified accidents caused by* Bothrops* snakes, 18% (52 cases) were caused by* B. pauloensis* [18], thus evidencing that the study of the venom of this snake species can be of medical relevance. In this work, we describe the isolation and enzymatic characterization of a highly basic PLA_2_ from the venom of* B. pauloensis. *We also investigated whether this isolated PLA_2_ possesses neurotoxic activity.

## 2. Material and Methods

### 2.1. Venom and Reagents

Venom was purchased from Sigma Chemical Co. (St. Louis, MO, USA).

Solvents (HPLC grade), 4-nitro-3-octanoyloxy-benzoic acid, sequencing grade bovine pancreatic trypsin and other reagents were also obtained from Sigma Chemical Co. (St. Louis, MO, USA).

### 2.2. Reverse Phase HPLC (RP-HPLC)

Bp-13 PLA_2_ from* B. pauloensis* venom was purified by reverse phase HPLC, according to the method described by Ponce-Soto et al. [19], with minor changes. Briefly, 5 mg of the whole venom was dissolved in 200 *μ*L of buffer A (0.1% TFA) and centrifuged at 4500 g; the supernatant was then applied to a *μ*-Bondapak C18 column (0.78 × 30 cm; Waters 991-PDA system), previously equilibrated in buffer A for 15 min. The protein elution was then conducted using a linear gradient (0–100%, v/v) of buffer B (66.5% acetonitrile in buffer A) at a constant flow rate of 1.0 mL/min. The chromatographic run was monitored at 280 nm of absorbance. The purity and PLA_2_ activity were monitored according to Sections 2.3 and 2.6. All fractions eluted were lyophilized and then stored at −20°C for further biochemical and pharmacological assays.

### 2.3. Electrophoresis

Tricine SDS-PAGE in a discontinuous gel and buffer system was used to estimate the molecular mass of Bp-13 PLA_2_, under reducing and nonreducing conditions [20]. The used molecular weight markers in kDa were phosphorylase B: ~ 94, albumin: 67, ovalbumin: 43, carbonic anhydrase: 30, soybean trypsin inhibitor: 20, and *α*-lactoalbumin: 14 (GE Healthcare).

### 2.4. Amino Acid Analysis

Amino acid analysis was performed on a Pico-Tag Analyzer (Waters Systems), as described by Heinrikson and Meredith [21], with minor changes. Bp-13 PLA_2_ sample (30 *μ*g) was hydrolyzed at 105°C for 24 hours, in 6 M HCl (Pierce sequencing grade) containing 1% phenol (w/v). Hydrolyzates were reacted with 20 *μ*L of derivatized solution (ethanol : triethylamine : water : phenylisothiocyanate, 7 : 1 : 1 : 1, v/v) for one hour at room temperature. Afterwards, PTC-amino acids were identified and quantified by HPLC, by comparing their retention times and peak areas with those from a standard amino acid mixture (Sigma-Aldrich).

### 2.5. Mass Spectrometry

Molecular mass of intact native and alkylated Bp-13 PLA_2_ was analyzed by MALDI-TOF mass spectrometry using a Voyager-DE PRO MALDI-TOF apparatus (Applied Biosystems, Foster City, CA, USA) equipped with a pulsed nitrogen laser (337 nm, pulse with 4 ns). The amount of 1 *μ*L of sample in 0.1% TFA was mixed with 2 *μ*L of sinapinic acid matrix (3, 5-dimethoxy-4-hydroxycinnamic acid). The matrix was prepared with 30% acetonitrile and 0.1% TFA and its mass analyzed under the following conditions: 25 kV accelerating voltage, the laser fixed at 2890 *μ*J/cm^2^, 300 ns delay, and linear analysis mode [22].

For de novo sequencing of N-terminal, the first 52 amino acids from Bp-13 PLA_2_, alkylated tryptic peptides were fractionated by RP-HPLC, manually collected, lyophilized, and resuspended in 80% H_2_O, 20% acetonitrile and 0.1% TFA. One peptide was introduced separately into the mass spectrometer source using a syringe pump at a 500 nl/min flow rate. Before performing a tandem mass spectrum, an ESI/MS mass spectrum (TOF MS mode) was acquired for each HPLC fraction over the mass range of 400–2000* m/z*, aiming to select the ion of interest. Subsequently, these ions were fragmented in the collision cell (TOF MS/MS mode). Different collision energies were used depending on the mass and charge state of the ions. The resulting product-ion spectra were acquired with the TOF analyzer and deconvoluted using the MassLynx-MaxEnt 3 algorithm (Waters). Singly charged spectra were manually processed using the PepSeq application included in MassLynx.

### 2.6. PLA_2_ Activity

PLA_2_ activity was measured using the assay described by Cho and Kézdy [23] and Holzer and Mackessy [24] modified for 96-well plates. The standard assay mixture contained 200 *μ*L of buffer (10 mM Tris–HCl, 10 mM CaCl_2_, and 100 mM NaCl, pH 8.0), 20 *μ*L of synthetic chromogenic substrate 4-nitro-3- (octanoyloxy) benzoic acid 3 mM, 20 *μ*L of water, and 20 *μ*L of PLA_2_ fractions (1 mg/mL) or whole venom (1 mg/mL) in a final volume of 260 *μ*L. After adding the samples, the mixture was incubated for up to 40 min at 37°C, absorbance reading at intervals of 10 min. The enzyme activity, expressed as the initial velocity of the reaction (*V*_0_), was calculated based on the increase of absorbance after 20 min.

The pH and optimal temperature of PLA_2_ were determined by incubating the four reaction buffers with different pH ranging from 4 to 10 and at different temperatures, respectively. The effect of substrate concentration (40, 20, 10, 5, 2.5, 1.0, 0.5, 0.3, 0.2, and 0.1 mM) on enzyme activity was determined by measuring the increase of absorbance after 20 min in optimum pH and temperature. The effect of different concentration of Ca^2+^ on Bp-13 PLA_2_ enzymatic activity was tested by preincubating the enzyme with different ion concentrations (0.005, 0.025, 0.05, 0.1, 0.2, 0.3, 0.4, and 0.5 M) at 37°C for 30 minutes prior to standard experiment. Also, the effects of different divalent ions (Sr^2+^, Mg^2+^, Mn^2+^, and Cd^2+^, 5 mM) were tested in presence (1 mM) or absence of Ca^2+^. Finally the effect of urea (4 M) on the enzymatic activity was tested by preincubating Bp-13 for 30 minutes at 37°C.

All assays were done in triplicate and the absorbance at 425 nm was measured using a VersaMax 190 multiwell plate reader (Molecular Devices, Sunnyvale, CA, USA).

### 2.7. BC, PND, and EDL Nerve-Muscle Preparations

Male Swiss mice (Mus musculus) weighing 20–30 g and 8-day-old young chick (Hy Line W36) were used for twitch-tension studies in presence of Bp-13 PLA_2_. Mice and young chick were sacrificed by halothane inhalation. The hemidiaphragm (PND) and extensor digitorum longus (EDL) muscles isolated from mice and biventer cervicis muscle (BC) isolated from chick were mounted according to Bülbring [25] and Ginsborg and Warriner [26], respectively.

The PND and EDL mammal preparations were suspended under a constant resting tension of 5 g/cm and 0.5 g/cm, respectively, in a 5 mL (for PND) and 3.5 mL (for EDL) organ bath containing aerated (95% O_2_-5% CO_2_) Tyrode solution, whose composition in mM was NaCl 137, KCl 2.7, CaCl_2_ 1.8, MgCl_2_ 0.49, NaH_2_PO_4_ 0.42, NaHCO_3_ 11.9, and glucose 11.1, pH 7.4, 37°C. A supramaximal pulse (0.1 Hz, 0.2 ms) delivered by a Grass S48 electronic stimulator (Grass Instrument Co., Quincy, MA, USA) was applied through electrodes placed around the motor phrenic nerve (PND) and tendon (EDL). For both preparations, the isometric muscle tension was recorded using a force-displacement transducer Load Cell BG 50 g (Kulite Semiconductor Products Inc., Leonia, NJ, USA) coupled to a physiograph (Gould RS 3400, Cleveland, OH, USA). The PND preparations were allowed to stabilize for at least 20 min before the addition of Bp-13 toxin at different concentrations: 0.71 *μ*M (10 *μ*g/mL), 1.42 *μ*M (20 *μ*g/mL), 3.56 *μ*M (50 *μ*g/mL), and 7.12 *μ*M (100 *μ*g/mL). The EDL preparations were incubated with Bp-13 toxin at 3.56 *μ*M.

Some curarized PND preparations (d-tubocurarine, 10 *μ*M) were incubated with Bp-13 toxin at 1.42 *μ*M and 3.56 *μ*M concentrations and the twitch response was recorded under direct muscle stimulation at supramaximal pulses of 70 V, 0.1 Hz at 2 ms duration. The effect of divalent ions, Mg^2+^, Mn^2+^, Sr^2+^, and Cd^2+^ (10 mM), on the Bp-13 PLA_2_ activity was done by replacement of Ca^2+^ (1.8 mM) in the nutritive Tyrode solution; replacement of Ca^2+^ by Sr^2+^ (4 mM) was also assayed. The effect of temperature (5 to 60°C) during 20 min was read at 425 nm.

The biventer cervicis (BC) preparations were suspended in a 5 mL organ bath containing Krebs solution (composition in mM: NaCl 118.6, KCl 4.69, CaCl_2_ 1.88, KH_2_PO_4_ 1.17, MgSO_4_ 1.17, NaHCO_3_ 25.0, and glucose 11.65), aerated with carbogen (95% O_2_-5% CO_2_) at 37°C. A bipolar platinum ring electrode was placed around the muscle tendon, within which run the motor nerve trunk. Field stimulation using a Grass S48 stimulator set at 0.1 Hz, 0.2 ms, and 4–6 V was applied and the muscle contractions and contractures were recorded isometrically via force-displacement transducer coupled to a physiograph. The muscle responsiveness to exogenously applied acetylcholine (ACh, 110 *μ*M) and KCl (13.4 mM) was recorded in the absence of field stimulation both prior to toxin addition and at the end of the experiment (120 min). The BC preparation was stabilized for at least 15 min before addition of Bp-13 at concentration of 3.56 (50 *μ*g/mL) and 7.12 *μ*M (100 *μ*g/mL). The results were compared with control BC preparations incubated with Krebs solution alone.

### 2.8. Statistical Analyses

Results were reported as mean ± SEM. Differences among means was assessed by one-way ANOVA and followed by Mann-Whitney test for comparison between two groups. Differences were considered statistically significant if *P* < 0.05.

## 3. Results

### 3.1. Purification and Characterization

**Figure 1:**
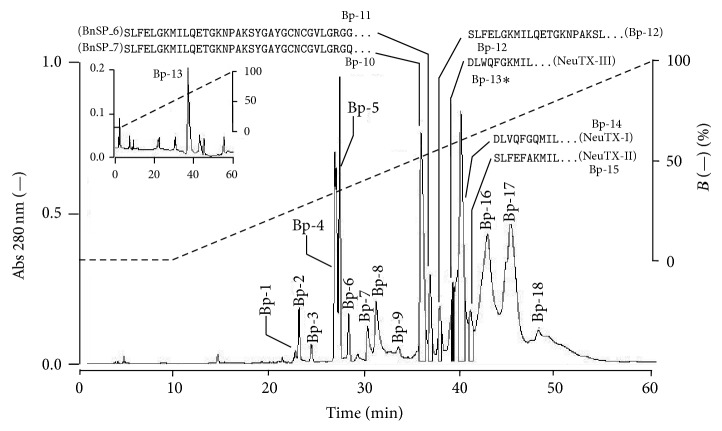
RP-HPLC chromatography of* Bothrops pauloensis* venom on *μ*-Bondapak C 18 column (0.78 cm × 30 cm; Waters 991-PDA system Waters). A sample of 20 mg from venom was eluted with solvent B (acetonitrile, 0–66%) at 25°C. The elution profile was monitored at 280 nm. The main fractions obtained are identified as Bp-1–Bp-18.

**Figure 2:**
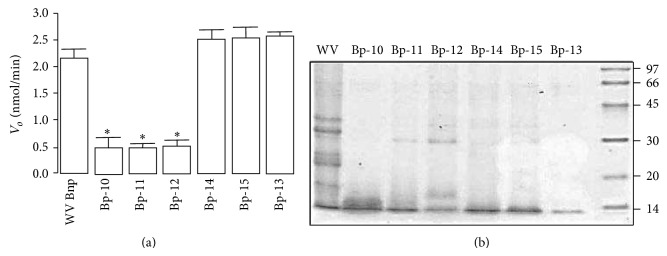
(a) PLA_2_ activity of* Bothrops pauloensis *whole venom (WV), Bp-13 PLA_2_. ANOVA and followed by Mann-Whitney test for comparison between two groups. Data are represented as mean ± SEM. (b) SDS-PAGE profile of Bp-13 PLA_2_, Mm, molecular mass markers (×10^−3^).

Fractionation of* B. pauloensis* venom by reverse phase HPLC (Figure 1) showed the elution of 18 main fractions: Bp-1 to Bp-18. The Bp-13, which was eluted at 39 min, was characterized as a not yet described PLA_2_-active toxin (Figure 2(a)). The purity of this peak was confirmed through rechromatography on an analytical RP-HPLC *μ*-Bondapak C18 column and showing a single peak (Figure 1, insert).

**Figure 3:**
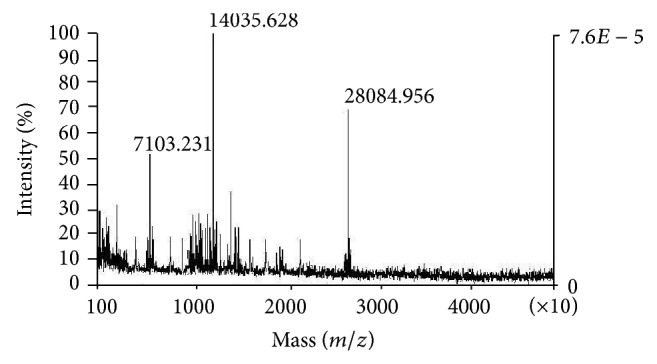
Mass determination of* Bothrops pauloensis* Bp-13 by MALDI-TOF mass spectrometry.

**Figure 4:**
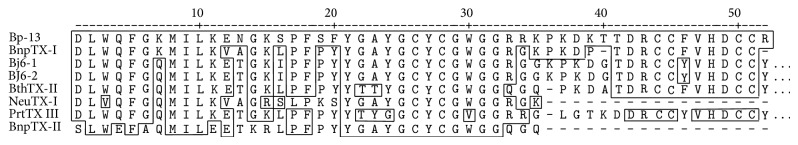
The amino acid sequence N-terminal, alignment of Bp-13 with selected PLA_2_ sequences obtained from the BLAST protein data bank (PubMed–Medline). PrtTX III from* Bothrops pirajai* [55], PLA_2_Bj p from* Bothrops jararacussu* [30], PLA_2_ 6-1 and PLA_2 _6-2 [22] and BthTx-II, bothropstoxin II, from* Bothrops jararacussu* [56], BnpTX-I and BnpTX-II [32], and NeuTX-I [12].

Tricine SDS-PAGE and MALDI-TOF mass spectrometry showed that Bp-13 PLA_2_ presented a molecular mass of ~14 kDa (Figure 2(b)) and 14035.628 Da (Figure 3), respectively. The amino acid analysis revealed the following composition: Asx/9, Glx/5, Ser/4, Gly/13, His/2, Arg/5, Thr/7, Ala/6, Pro/11, Tyr/14, Val/4, Cys/14, Ile/3, Leu/11, Phe/3, and Lys/12 with a high content of basic and hydrophobic amino acids and 14 half-cystine residues. Its N-terminal sequence of the 52 initial residues was as follows: DLWQFGKMIL KENGKSPFSF YGAYGCYGGW GGRRKPKDKT TDRCCFVHDC CR. Such amino acid sequencing of Bp-13 PLA_2_ shares 95% sequence identity with other bothropic PLA_2_s (Figure 4).

### 3.2. Enzymatic Characterization of Bp-13 PLA_2_

**Figure 5:**
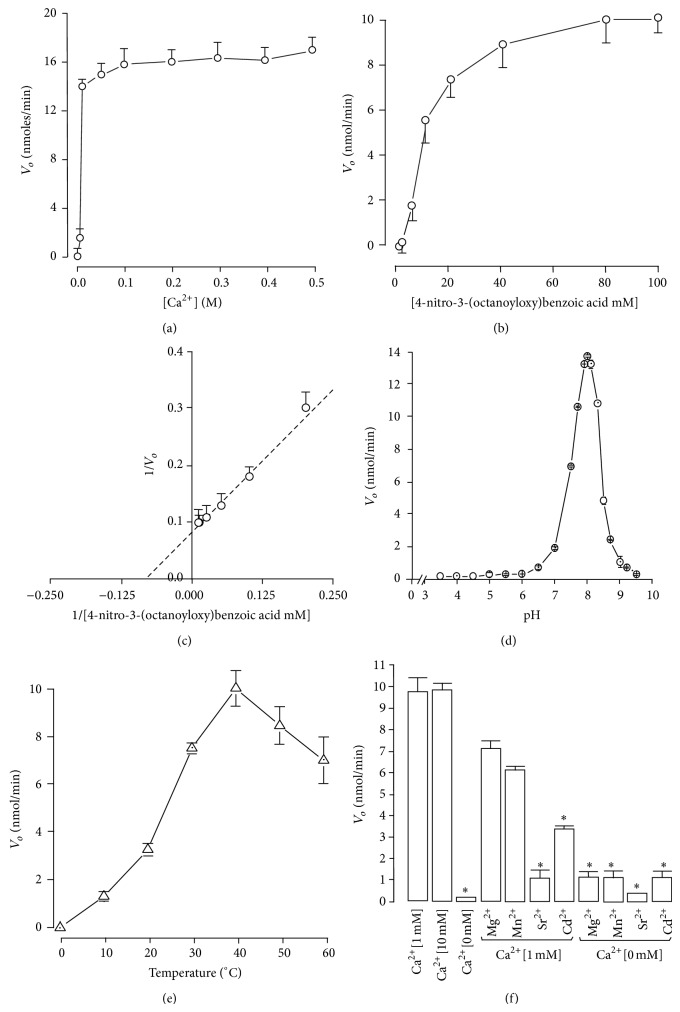
Kinetic analysis of Bp-13 PLA_2_ activity. (a) Influence of calcium ion on PLA_2_ activity; (b) effect of substrate concentration on the kinetics of Bp-13 PLA_2_; (c) Lineweaver-Burk (double-reciprocal) plot of Bp-13 PLA_2_; (d) optimal pH for PLA_2_ activity; (e) optimal temperature for PLA_2_ activity; (f) influence of ions (10 mM each) on PLA_2_ activity in the absence or presence of 1 mM and 10 mM Ca^2+^. The results are the mean ± SEM of five experiments. ^*^ *P* < 0.05 when compared with control values. ANOVA and followed by Mann-Whitney test for comparison between two groups.

The Bp-13 PLA_2_ activity measured through synthetic substrate 4-nitro-3-octanoyloxy-benzoic acid and 1–20 mM Ca^2+^ showed that the catalytic activity was expressed after 2 mM Ca^2+^, but the maximal PLA_2_ activity was reached with 10 mM Ca^2+^ (Figure 5(a)). For the conditions tested, Bp-13 PLA_2_ showed a discrete allosteric-like behavior, mainly at low substrate concentrations (Figure 5(b)). Estimated *V*_max⁡_ was 11.6 nmol/min/mg and *K*_*m*_ was 11.8 mM (Figure 5(c)). The optimal pH and temperature for development of the maximum enzymatic activity were 8.3 (Figure 5(d)) and ~38°C (Figure 5(e)), respectively. The addition of Mn^2+^, Mg^2+^, Sr^2+^, and Cd^2+^ (10 mM) in the presence of low Ca^2+^ concentration (1 mM) decreased enzyme's activity; the substitution of Ca^2+^ by Mn^2+^, Mg^2+^, Sr^2+^, or Cd^2+^ (10 mM) in the absence of Ca^2+^ (0 mM) also reduced PLA_2_ activity (Figure 5(f)). In addition, preincubation with urea (4 M) did not affect significantly the enzymatic activity of Bp-13 (data not shown).

### 3.3. Neuromuscular Activity

**Figure 6:**
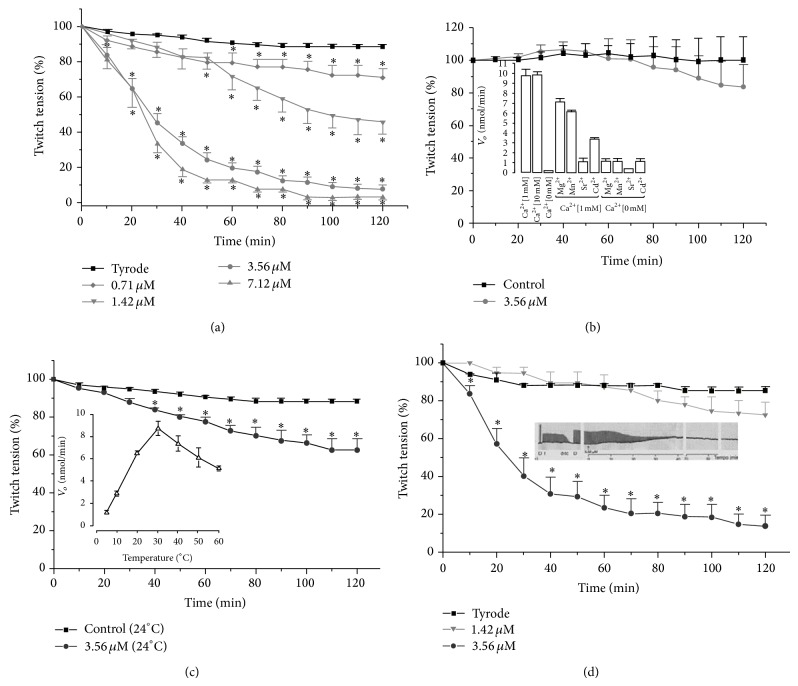
Twitch-tension response of direct and indirect stimulated PND preparations for 120 min. (a) The preparations were incubated with Tyrode (control) or Bp-13 PLA_2_ (0.71–7.12 *μ*M) at 37°C (*n* = 3–6 experiments); (b) twitch-tension response of PND preparations incubated with Bp-13 PLA_2_ in Tyrode solution and in Tyrode whose 1.8 mM Ca^2+^ was replaced by 4 mM Sr^2+^ at 37°C (*n* = 4–6); (c) twitch-tension response of indirectly stimulated PND preparations incubated with Tyrode (control) or Bp-13 (3.56 *μ*M) at 24°C (*n* = 4–6 experiments); (d) twitch-tension response of directly stimulated PND preparations incubated with Tyrode (control) or Bp-13 (1.42 and 3.56 *μ*M) at 37°C (*n* = 4–6 experiments). Twin arrows represent the time of toxin addition. Each point represents the mean ± SEM; ^*^ *P* < 0.05 compared to control values. ANOVA and followed by Mann-Whitney test for comparison between two groups.

Assays to study the neuromuscular activity of Bp-13 PLA_2_ were performed using avian BC preparation and rodent's PND and EDL preparations. The toxin induced a time- and concentration-dependent and irreversible twitch-tension blockade. In the PND preparations, the time needed for 50% paralysis in response to 7.12 *μ*M (*n* = 3), 3.56 *μ*M (*n* = 6), and 1.42 *μ*M (*n* = 3) of Bp-13 PLA_2_ was 18 ± 1 min, 28 ± 3 min, and 120 ± 4 min, respectively (*P* < 0.05); Bp13 at 0.71 *μ*M concentration induced a 25% paralysis only after 120 ± 2 min relative to control (*P* < 0.05) (Figure 6(a)). The catalytic activity of Bp-13 PLA_2_ was similar both in the presence of 1 or 10 mM in the nutritive bath of PND preparation. The addition of Mg^2+^, Mn^2+^, Sr^2+^, and Cd^2+^ (10 mM) in the nutritive Tyrode solution in the absence of Ca^2+^ or presence of 1 mM Ca^2+^ showed significant loss of the catalytic activity of the toxin indicating that these divalent ions cannot replace the Ca^2+^ for the development of the PLA_2_ catalytic activity. The replacement of 1.8 mM Ca^2+^ by 4 mM Sr^2+^ in the Tyrode solution prevented the blocking effect of Bp13 PLA_2_ (3.56 *μ*M) since the twitch-tension response showed an amplitude of 83.7 ± 14% after 120 min incubation which was not different from baseline of the control preparations (Figure 6(b)). The finding indicates that the neuromuscular blocking effect of the Bp-13 is calcium-dependent.

The effect of temperature (5°C–60°C) on the catalytic activity of Bp-13 PLA_2_ showed that the optimal enzymatic activity occurred around 38°C (Figure 6(c), insert). The neuromuscular blockade was prevented when the temperature incubation was set at 24°C; after 120 min, the twitch-tension response was 37.4% compared with the 90% seen at 37°C, Figure 6(c). The PND preparations previously treated with d-Tc (10 *μ*M) and under direct electrical stimulation showed that Bp-13 (1,42 and 3.56 *μ*M) was able to cause a significant contracture followed by blockade of the contractile response (73 ± 7% and 14 ± 6%, respectively, *n* = 3–6, *P* < 0.05, Figure 6(d)).

**Figure 7:**
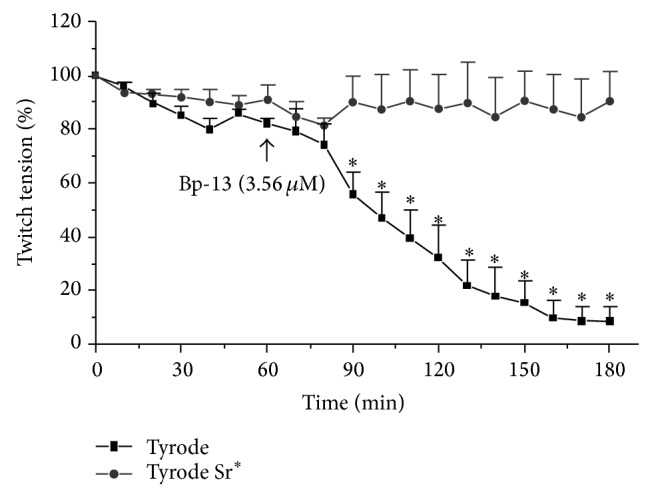
Twitch-tension response of indirectly stimulated EDL preparations incubated with 3.56 *μ*M of Bp-13 in Tyrode and Tyrode Sr^2+^. Each point represents the mean ± SEM of 3–6 experiments; ^*^ *P* < 0.05 compared to control values. ANOVA and followed by Mann-Whitney test for comparison between two groups.

In the EDL preparations, the time needed for 50% paralysis at a 3.56 *μ*M Bp13 PLA_2_ concentration was 120 min ± 2 min (Figure 7). As displayed in the Figure 7, the contractile response of the EDL preparation was maintained steady during the 60 min period, regardless of whether the EDL was incubated in normal Tyrode solution or in Tyrode solution whose Ca^2+^ (1.8 mM) was replaced by Sr^2+^ (4 mM). The Bp-13 (3.56 *μ*M) addition caused a significant blockade of the twitch tension which achieved 90% after 80 min of toxin addition in the normal Tyrode solution. The replacement of 1.8 mM Ca^2+^ by 4 mM Sr^2+^ also prevented completely such neuromuscular blockade induced by the toxin (3.56 *μ*M) which was sustained until the end of observation (180 min, *n* = 6, *P* < 0.05). Similarly, the lowering of temperature to 24°C prevented the blockade of twitch tension in the EDL preparation (not shown).

**Figure 8:**
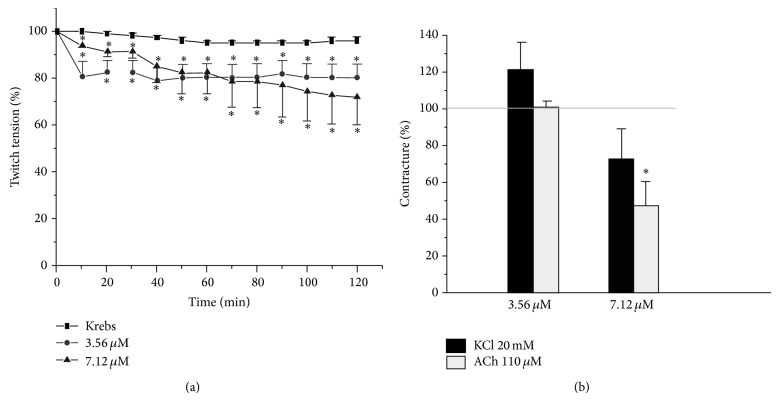
Twitch-tension response curve of indirectly stimulated BC preparations incubated with 3.56 and 7.12 *μ*M (50 and 100 *μ*g/mL, resp.) at 37°C. (a) Each point represents the mean ± SEM of 3–6 experiments. (b) Percentage of Ach (110 *μ*M) and KCl (20 mM) evoked contracture after Bp-13 administration; ^*^ *P* < 0.05 compared to control values. ANOVA and followed by Mann-Whitney test for comparison between two groups.

In relation to avian preparations, it was shown that they exhibited lower sensibility to Bp-13 PLA_2_ than the mammalian preparations. Bp-13 PLA_2_ (3.56 and 7.12 *μ*M) induced an irreversible but mild decrease of the twitch tension of 21 ± 6% and 28 ± 2% after 120 min, respectively (Figure 8(a)). The response to acetylcholine (ACh, 110 *μ*M) was significant just when Bp-13 PLA_2_ concentration was set at 7.12 *μ*M (*P* > 0.05). In contrast, Bp-13 PLA_2_ regardless of the concentration, 3.56 or 7.12 *μ*M, did not interfere in the KCl (13.4 mM) induced contracture (Figure 8(b)).

## 4. Discussion

The presence of Bp-10 and Bp-11 (K49 PLA_2_ homologous Bnsp 6 and Bnsp7) [8], Bp-14 and Bp-15 (Asp49 PLA_2_ NeuTX-I and NeuTX-II) [12], and Bp-12 (Lys49 PLA_2_) [11] has been already demonstrated in the* B. pauloensis *snake crude venom. Interestingly, another Asp49 PLA_2_, the Bp13, of the same* Bothrops *species venom was now isolated and characterized biochemically and pharmacologically. Such a diversity of PLA_2_ isoforms in the venom of a same species evidences the necessity of developing efficient methodologies to purify and identify different isoforms in venom fractions otherwise considered homogeneous. The RP-HPLC was more suitable to purify Bp-13 PLA_2_ than other conventional methods previously described for other toxins of the same venom, since it required just a single chromatographic step [19, 22, 27].

The purity of Bp-13 PLA_2_ was confirmed by rechromatography on an analytical RP-HPLC *μ*-Bondapak C18 column. SDS-PAGE showed the monomeric nature of Bp-13 and a relative molecular mass of ~14 kDa and it was confirmed by MALDI-TOF mass spectrometry with a molecular mass of 14035.628 Da. MALDI-TOF/MS has a precision in measuring protein molecular mass of 0.1% Da; thus, this characteristic allows us to demonstrate that Bp13 is another PLA_2_ isoform present in the* Bothrops pauloensis* venom, as was for other bothropic PLA_2_ isoforms [19, 22, 27–32].

The amino acid composition of Bp-13 PLA_2_ suggests that this PLA_2_ is a basic protein because it possesses more basic residues (Arg, His, and Lys, total 19) than acid residues (Asx/Gnx, total 13), 14 half- Cys, and also because this protein showed high content of residues Tyr, Pro, Gly, and Lys which was a composition featured by other catalytic active bothropic myotoxins such as the 6-1 PLA_2_ and 6-2 PLA_2_ isoforms from* B. jararacussu *orthe BaTX, a basic PLA_2_ from* B. alternatus* [22, 27].

In Asp49 PLA_2_, a conserved N-terminal helix region forms a hydrophobic channel involving L2, Q4, F5, and I9. Conversely, the level of identity between PLA_2_s is very high in Ca^2+^-loop sequence (residues 24–34 YGCXCGXGGRG) and in the active site (residues 42–54 DRCCFVHDCCYXK) [22, 33–35]. The conserved residues Y28, G30, G32, Asp49, H48, and Y52 are directly or indirectly linked to Bp-13 PLA_2_ catalysis. The N-terminal amino acid sequence of the first 52 residues from Bp-13 PLA_2_ shows these regions highly conserved and directly linked to catalytic activity.

Bp-13 PLA_2_ herein analyzed showed the presence of some important mutations in N-terminal sequence (up to the 52nd residue). Thus, Bp-13 PLA_2_ shows K7 -> Q7, N13 -> T13, F20 -> Y20, R34 -> G34, and F46 -> Y46, which are strategic positions for expression of the catalytic activity. The presence of K7 in Bp-13 PLA_2_ shows that this residue can contribute to keep hydrophobic cavity conformation of the N-terminal region. The N-terminal channel present in PLA_2_ enzymes is highly conserved and provides access to the lipid substrate to the PLA_2_ catalytic site. Also, Bp-13 shows at position 13 the lack of Thr residue usually found in other PLA_2_s enzymes; however, both BnpTX-I (A13 -> T13) and NeuTx-I (A13 -> T13) also showed mutation in this position for polar residue, suggesting that this position is not as well conserved and that it could be a structural feature for the PLA_2_ of* B. pauloensis*. The same was observed for the position 16, but in this particular case this position conserved its hydrophobic nature. Despite the change, Bp-13 still maintains catalytic activity and indicates that these residues have no important role in Bp-13 activity [33, 36].

Breithaupt [37] reported that* Crotalus* PLA_2_ shows classic Michaelis-Menten behavior. However, PLA_2_ activity of Bp-13 from* B. pauloensis* shows a discrete allosteric-like behavior, and this activity is enhanced by the presence of even low Ca^2+^ concentrations. The PLA_2_ from* C. durissus terrificus* venom exhibits a typical PLA_2_ activity, since it hydrolyzes synthetic substrates at position 2 and preferentially attacks substrates in their micellar state [24, 37]. Similarly, Bp-13 PLA_2_ exhibits allosteric behavior with a *V*_max⁡_ of 11.6 nmol/min and a *K*_*m*_ of 11.8 mM.

However, at low concentrations of the synthetic substrate, Bp-13 PLA_2_ showed a sigmoidal kinetic behavior; this phenomenon was observed for other Crotalinae PLA_2_s [2, 22, 38–40], suggesting an allosteric behavior for these enzymes. The allosteric term was originally used for enzymes with altered kinetic properties in the presence of ligands (effectors) that do not show any structural similarity to the substrate. Allosteric enzymes show a number of properties that distinguish them from the nonallosteric ones. Sigmoidal kinetics in the velocity substrate curve, the existence of effectors, and a polymeric structure are some of the properties of a genuine allosteric enzyme. In the case of Crotalinae D49-PLA_2_, SDS-PAGE without reducing agents showed a weak band at ~28 kDa [2, 40] indicating that some molecule populations of these enzymes exist in a dimeric form, which could be responsible for the observed enzymatic behavior.

Bp-13 PLA_2_ was resistant to heat and acid like PLA_2_s from* Crotalus d. cascavella* [38, 41],* C. d. collilineatus*, and* Lachesis muta muta* [42] venoms; Bp-13 optimal activity was at pH 8.3 and was inactivated at pH higher than 9, like the PLA_2_ from* C. mitchelli pyrrhus* [24],* C. d. terrificus* [43], and* B. neuwiedi* [44] snake venoms. The maximal enzymatic activity occurred at ~38°C and persisted at 60°C, indicating a heat-stable enzyme.

Pharmacologically, as for other types of PLA_2_, the activity of Bp-13 PLA_2_ was shown to be completely Ca^2+^-dependent [45]. The coincubation of Bp-13 PLA_2_ with other divalent ions (Mg^2+^, Mn^2+^, Sr^2+^, and Cd^2+^) in the presence of 1 mM Ca^2+^ or in Ca^2+^ absence reduced or abolished the enzymatic activity.

The Ca^2+^ replacement by 4 mM Sr^2+^ abolished the neuromuscular blockade. It is well known that calcium ions are essential cofactors for the enzymatic activity of both toxic and nontoxic phospholipases A_2_ [46]. Several divalent ions, including Sr^2+^, can bind to the same site of the Ca^2+^, allowing neuromuscular transmission; nevertheless, this ion does not substitute Ca^2+^ in the catalysis processes [47–50]. Thus, the observation that neuromuscular effect of Bp-13 was Ca^2+^-dependent indicates that the enzymatic activity might contribute for such effect as for neuwieditoxin I and neuwieditoxin II, an Asp49 PLA_2_ from* B. pauloensis* [12]. Also, the lowering of the temperature of incubation bath to 24°C abolishing the neuromuscular effect of Bp-13 indicates that the enzymatic activity has a role on the neuromuscular action in PND and EDL preparations. Such finding is similar to the one observed by Galbiatti et al. [14] with Bmaj-9 PLA_2_ from* B. marajoensis* venom, also an Asp49 PLA_2_. Likewise, a high catalytic activity was found in neuwieditoxin-I and -II Asp49 PLA_2_s, whose neuromuscular blockade was also temperature-dependent [12]. Both the dependence of Ca^2+^ and the need of temperature equal or above 30°C for enzymatic activity of Bp-13 identify the toxin as a typical Asp49 PLA_2_.

Bothropic envenomation does not cause neurotoxic clinical signs, but some experimental studies have shown that the venom of several species causes neuromuscular blockade* in vitro* [8, 11–14, 32, 51, 52] and induces peripheral muscular weakness signs [6, 53].

Borja-Oliveira et al. [54] reported that* B. pauloensis* crude venom causes partial blockade of directly evoked muscular contractions in BC preparations; in 2007, the authors suggested that neuwieditoxin-I and -II (Asp49 PLA_2_s) from* B. pauloensis* venom were probably responsible for the venom presynaptic neurotoxicity* in vitro*. However, herein it was shown that BC preparations showed low responsiveness to the Bp-13 (less than 30% of neuromuscular blockade) when compared to the crude venom. This could mean that this new Asp49 PLA_2_ from* B. neuwiedi* venom has preponderantly a muscular action, thus differing from the presynaptic neurotoxic action of neuwieditoxin-I and -II Asp49 PLA_2_s referred by Borja-Oliveira et al. [12].

Taken together, these results identify Bp-13 isolated from* Bothrops pauloensis* snake venom, as a new member of the Asp49 PLA_2_ family. It is suggested that the Bp-13 PLA_2_ catalytic activity may contribute to the neuromuscular effect already reported for the crude venom on rodent preparations. It is suggested that the main effect of the Bp-13 toxin seems to be on the fiber sarcolemma, with prominence in the mice preparation. In BC preparations, the Bp-13 PLA_2_ showed little neuromuscular effect. This study is an additional contribution for understanding the toxic effect caused by the* Bothrops pauloensis* crude venom.
